# Efficient multiparty quantum key agreement with collective detection

**DOI:** 10.1038/s41598-017-15227-6

**Published:** 2017-11-10

**Authors:** Wei Huang, Qi Su, Bin Liu, Yuan-Hang He, Fan Fan, Bing-Jie Xu

**Affiliations:** 1 Science and Technology on Communication Security Laboratory, Chengdu, 610041 China; 2 State Key Laboratory of Cryptology, Beijing, 100878 China; 3 College of Computer Science, Chongqing University, Chongqing, 400044 China

## Abstract

As a burgeoning branch of quantum cryptography, quantum key agreement is a kind of key establishing processes where the security and fairness of the established common key should be guaranteed simultaneously. However, the difficulty on designing a qualified quantum key agreement protocol increases significantly with the increase of the number of the involved participants. Thus far, only few of the existing multiparty quantum key agreement (MQKA) protocols can really achieve security and fairness. Nevertheless, these qualified MQKA protocols are either too inefficient or too impractical. In this paper, an MQKA protocol is proposed with single photons in travelling mode. Since only one eavesdropping detection is needed in the proposed protocol, the qubit efficiency and measurement efficiency of it are higher than those of the existing ones in theory. Compared with the protocols which make use of the entangled states or multi-particle measurements, the proposed protocol is more feasible with the current technologies. Security and fairness analysis shows that the proposed protocol is not only immune to the attacks from external eavesdroppers, but also free from the attacks from internal betrayers.

## Introduction

Since the pioneer work published by Bennett and Brassard in 1984^1^, the principles of quantum mechanics was introduced to protect information security, which opened the new research area of cryptography, quantum cryptography^1–45.^ Thus far, many branches of quantum cryptography have been presented to offer various security properties, including quantum key distribution (QKD)^1–12,^ quantum secure direct communication (QSDC)^13–20,^ quantum secret sharing (QSS)^21–26,^ quantum secure multiparty computation(QSMC)^27–41^, etc. Recently, a new branch of quantum cryptography, quantum key agreement (QKA), has received more and more attention. Different from QKD, in which a participant generates the secret key and then distributes it to the others, each of the participants in the QKA protocol should contribute his/her influence to the final common key. In other words, aside from having the ability of resisting the external attackers from stealing the key as QKD protocols, QKA protocols should also have the ability of being immune to the participant attack, i.e., a non-trivial subset of the participants tries to predetermine the final common key alone.

As the earliest and the maturest branch of quantum cryptography, QKD has been developed theoretically and experimentally to be one of the most promising technologies in quantum cryptography. However, as a new branch of quantum cryptography, QKA is still in its infancy. In 2004, the first QKA protocol was proposed by Zhou *et al*. with the maximally entangled states and the technique of quantum teleportation^46^. Almost simultaneously, Hsueh and Chen proposed another QKA protocol by employing the maximally entangled states^47^. However, Tsai *et al*. demonstrated that neither of the two protocols could qualify as a QKA protocol^48,49^, i.e., neither of them can simultaneously achieve the fairness property or security property. In 2010, by utilizing the ideas of the famous BB84 protocol, Chong *et al*. proposed a QKA protocol based on the technique of delayed measurement^50^. In 2011, in order to close the flaws pointed out by Tsai *et al*.^49^, Chong *et al*.^51^ presented a modified version of the QKA protocol proposed in ref.^47^. In 2014, Huang *et al*. presented two QKA protocols which can be immune to the collective noises^52,53^, In 2016, He *et al*. put forward a QKA protocol by utilizing four-particle entangled states^49^. Nevertheless, all of the protocols mentioned above are limited to the two-party case^46–54^.

To extend the research of QKA to the multiparty case, researchers begin to focus on MQKA, where more than two participants cooperate to share a common key securely and fairly. In 2013, by utilizing EPR pairs and the technique of entanglement swapping, Shi *et al*.^55^ tried to propose the first MQKA protocol. However, Liu *et al*.^56^ pointed out that this protocol failed to achieve the fairness property, and further put forward a distributed-mode MQKA protocol with single photons. In 2013, Sun *et al*.^57^ made the attempt to improve the efficiency of Liu *et al*.’s MQKA protocol and propose a MQKA protocol in travelling mode. Unfortunately, this protocol has also been demonstrated to be unfairness^58^. In 2014, a distributed-mode MQKA protocol is proposed with GHZ states by Xu *et al*.^59^. In the same year, two travelling-mode MQKA protocols were presented with cluster states and six-qubit states, respectively by Sun *et al*.^60,61^. Meanwhile, Shukla *et al*. put forward a travelling-mode MQKA protocol by employing Bell state and Bell measurements^62^. Nevertheless, Zhu *et al*. found out that there exist some loopholes in Shukla *et al*.’s protocol and further proposed an improved version of this protocol^63^. In 2016, Sun *et al*. presented an MQKA protocol based on commutative encryption^64^. However, these travelling-mode MQKA protocols^60,61,63,64^ are unfair under the collusion attack from internal betrayers^65,66^. In other words, a non-trivial subset of the involved participants can conspire to predetermine the final common key without being noticed by others. In 2016, Huang *et al*. proposed a travelling-mode MQKA protocol with single photons and unitary operations^67^. Recently, Cao *et al*. also presented a travelling-mode MQKA protocol based on quantum search algorithm^68^.

In a travelling-mode MQKA protocol, every quantum information carrier sequence, which is used for encoding secret information, will sequentially be processed by all the involved participants of the protocol. That is to say, in an $n$-party QKA protocol, each quantum information carrier sequence will be transmitted $n$ times. If the eavesdropping is checked after every transmission, a lot of quantum states (usually referred to the decoy qubits) need consuming, and hence the qubit efficiency and measurement efficiency will drop substantially. To our best knowledge, all of the existing travelling-mode MQKA protocols have fallen into this category^57,60–64,67^, i.e., eavesdropping check is needed in each transmission of the quantum information carriers sequence. To improve the qubit efficiency of travelling-mode MQKA, we devote ourselves to designing a travelling-mode MQKA protocol where the eavesdropping check is not required in each transmission of the quantum information carrier sequence. In this paper, we propose an efficient travelling-mode MQKA with single photons, inspired by the results of refs^11,12,64,67^. By employing the ideas of collective eavesdropping detection strategy^11,12^ which was first proposed by Shih *et al*.^11^, the number of eavesdropping checks needed in this protocol has been significantly reduced. Hence, the qubit efficiency and measurement efficiency of the proposed protocol are higher than those of the existing ones (including both the protocols in travelling mode and the ones in distributed mode) in theory. In addition, compared with the protocols which utilize the entangled states or multi-particle measurements, the proposed protocol is more feasible with the current technologies due to the utilization of single photons and single-particle measurements.

The remainder of this paper is organized as follows. Next section first presents our travelling-mode MQKA protocol with single photons in detail. Then the security and fairness of the proposed protocol is analyzed. Finally, a discussion as well as a brief conclusion is given in the “Discussion section”.

## Results

### The proposed travelling-mode MQKA protocol

Herein we propose a new travelling-mode MQKA protocol by employing single photons, where $n$ participants, ${P}_{1}$, ${P}_{2}$, …, ${P}_{n}$ cooperate to establish a common key securely and fairly. For each of the involved participant ${P}_{i}$, 1 $\le$ $i$ $\le$ $n$, we suppose that he/she has an ($l$ + $k$ $l$)-bit private random key ${K}_{i}$ and n−1($l$ + $k$ $l$)-bit random controlling strings ${C}_{i(i+\mathrm{1)}}$, ${C}_{i(i+\mathrm{2)}}$, …, ${C}_{i(i-\mathrm{1)}}$, where ${C}_{i(j+n)}$ = ${C}_{ij}$, and $k$ is the detection rate. Similar to the existing QKA protocols^46–67^, the classical communication channels are assumed to be authenticated in this protocol. Moreover, the technique of “block transmission”^13–16^ where the quantum information carriers are ordered and transmitted in blocks, is utilized to ensure the security of the photon transmission in this protocol. The detailed steps of the proposed MQKA protocol can be described as follows.

(1) Each of the $n$ participants ${P}_{i}$ ($i$ = 1, 2, …, $n$) prepares an ordered sequence (denoted as ${S}_{i}$) of $l$ + $kl$ single photons, each of which is randomly in one of the four states in {$\mathrm{|0}\rangle$, $\mathrm{|1}\rangle$, $|+\rangle$, $|-\rangle$}. Here,

1 $$|+\rangle =\frac{1}{\sqrt{2}}\mathrm{(|0}\rangle +\mathrm{|1}\rangle ),\quad \quad |-\rangle =\frac{1}{\sqrt{2}}\mathrm{(|0}\rangle -\mathrm{|1}\rangle \mathrm{)}.$$

Then ${P}_{i}$ sends ${S}_{i}$ to ${P}_{i+1}$, for $i$ = 1, 2, 3, …, $n$, where ${P}_{n+i}$ = ${P}_{i}$.

(2) After the reception of ${S}_{i}$, ${P}_{i+1}$ announces the fact. Then he/she encodes his private key and the corresponding controlling binary string, ${K}_{i+1}$ and ${C}_{(i+\mathrm{1)}i}$, onto ${S}_{i}$. Specifically, if the $j$-th bits of ${K}_{i+1}$ and ${C}_{(i+\mathrm{1)}i}$, i.e., ${K}_{i+1}^{j}$ ${C}_{(i+\mathrm{1)}i}^{j}$, are 00/10/01/11, ${P}_{i+1}$ performs the unitary operation $I$/$F$/$W(\frac{p\pi }{n})$/$F$ $W(\frac{p\pi }{n})$ on the $j$-th photon of ${S}_{i}$ (i.e., ${S}_{i}^{j}$), for $j$ = 1, 2, 3, …, $l$ + $k$ $l$, where

2 $$\begin{array}{c}I=(\begin{array}{cc}1 & 0\\ 0 & 1\end{array}),\quad \quad F=-iY=-i(\begin{array}{cc}0 & -i\\ i & 0\end{array})=(\begin{array}{cc}0 & -1\\ 1 & 0\end{array}),\\ W(\frac{p\pi }{n})={e}^{\frac{-p\pi Y}{2n}}=\,\cos (\frac{p\pi }{2n})I-i\,\sin (\frac{p\pi }{n})Y=(\begin{array}{cc}\cos (\frac{p\pi }{2n}) & -\,\sin (\frac{p\pi }{2n})\\ \sin (\frac{p\pi }{2n}) & \cos (\frac{p\pi }{2n})\end{array}),\end{array}$$

where $p$ is a prime number, and $p$ $<$ $n$. The effect of $F$ and $W(\frac{p\pi }{n})$ on the four polarized photons, $\mathrm{|0}\rangle$, $\mathrm{|1}\rangle$, $|+\rangle$ and $|-\rangle$, can be respectively described as

3 $$\begin{array}{c}F\mathrm{|0}\rangle =\mathrm{|1}\rangle ,\quad F\mathrm{|1}\rangle =-\mathrm{|0}\rangle ,\quad F|+\rangle =-|-\rangle ,\quad F|-\rangle =|+\rangle ,\\ W(\frac{p\pi }{n})\mathrm{|0}\rangle =\,\cos (\frac{p\pi }{2n})\mathrm{|0}\rangle +\,\sin (\frac{p\pi }{2n})\mathrm{|1}\rangle ,\\ W(\frac{p\pi }{n})\mathrm{|1}\rangle =\,\cos (\frac{p\pi }{2n})\mathrm{|1}\rangle -\,\sin (\frac{p\pi }{2n})\mathrm{|0}\rangle ,\\ W(\frac{p\pi }{n})|+\rangle =\,\cos (\frac{p\pi }{2n})|+\rangle -\,\sin (\frac{p\pi }{2n})|-\rangle ,\\ W(\frac{p\pi }{n})|-\rangle =\,\cos (\frac{p\pi }{2n})|-\rangle +\,\sin (\frac{p\pi }{2n})|+\rangle .\end{array}$$

After finishing performing the unitary operations, ${P}_{i+1}$ sends the new sequence (denoted as ${S}_{i(i+\mathrm{1)}}$) to ${P}_{i+2}$. Meanwhile, each of the other $n$−1 participants processes his/her received sequence just in the same way and sends the obtained new sequence to next participant. That is, ${P}_{i+1}$ sends ${S}_{i(i+\mathrm{1)}}$ to ${P}_{i+2}$, for $i$ = 1, 2, 3, …, $n$. Here, we denote what the $n$ participants have done in this step as the first round of encoding (denoted as Round 1).

(3) The $n$ participants execute another $n$−2 rounds of encoding similar to the process of Round 1. That is, just in the same way as ${P}_{i+1}$ dose in steps (2), the $n$ participants repeatedly execute information encoding phase until ${P}_{i}$ receives ${S}_{i(i+\mathrm{1)...(}i-\mathrm{1)}}$ from ${P}_{i-1}$, for $i$ = 1, 2, 3, …, $n$. In other words, for each participant ${P}_{i}$, $i$ = 1, 2, 3, …, $n$, when he/she receives the photon sequence from ${P}_{i-1}$ in Round $v$, $v$ $\in$ $\mathrm{\{2}$, …, $n$−1$\}$, he/she encodes ${K}_{i}$ and ${C}_{i(i+n-v)}$(=${C}_{i(i-v)}$) onto the received sequence. Afterwards, he/she sends the processed sequence ${S}_{(i-v)(i-v+\mathrm{1)..}.i}$ to ${P}_{i+1}$. This procedure is repeated until ${P}_{i}$ receives ${S}_{i(i+\mathrm{1)...(}i-\mathrm{1)}}$ from ${P}_{i-1}$.

(4) Upon receiving ${S}_{i(i+\mathrm{1)...(}i-\mathrm{1)}}$ from ${P}_{i-1}$, ${P}_{i}$ announces the fact. Once conforming that every participant has received the photon sequence prepared by himself/herself, all the $n$ participants cooperate to randomly choose $kl$ positions (from $l$ + $kl$ positions), at which the quantum information carriers will be used to check eavesdropping hereafter. Concretely, ${P}_{1}$ randomly chooses $\lfloor \frac{kl}{n}\rfloor$ positions, and publishes the information of each chosen position, where $\lfloor x\rfloor$ represents the largest one of the integers which are smaller than $x$. Then ${P}_{2}$ chooses another $\lfloor \frac{kl}{n}\rfloor$ positions from the remaining $kl$−$\lfloor \frac{kl}{n}\rfloor$ positions and announces the position information. Just in the same way, ${P}_{i}$ ($i$ = 3, 4, …, $n$−1) randomly chooses $\lfloor \frac{kl}{n}\rfloor$ from the remaining $l$−($i$−1)$\lfloor \frac{kl}{n}\rfloor$ positions and publishes the position information. It should be noted that ${P}_{n}$ should choose $kl$−($n$−1)$\lfloor \frac{kl}{n}\rfloor$ positions for this check in order to make sure that the total number of the positions chosen by them is $kl$.

(5) After the participants finishing choosing the positions used for checking eavesdropping, each participant announces his/her $n$−1 controlling strings. Namely, ${P}_{i}$ announces ${C}_{ij}$, for $i$ = 1, 2, 3, …, $n$, and $j$ = $i$ + 1, $i$ + 2, …, $i$−1. With the announced information, each of the participants deduces a ($l$ + $kl$)-bit string as follows. ${P}_{i}$ ($i$ = 1, 2,…, $n$) first offsets the effect of the controlling operations (operations determined by the bits of controlling strings) that have been performed on the photons of ${S}_{i(i+\mathrm{1)...(}i-\mathrm{1)}}$. Concretely, to offset the controlling operations performed on the photons of ${S}_{i(i+\mathrm{1)...(}i-\mathrm{1)}}$, ${P}_{i}$ performs $W(\frac{-{\tilde{C}}_{i}^{j}p\pi }{n})$ on the $j$-th photon of ${S}_{i(i+\mathrm{1)...(}i-\mathrm{1)}}$ (i.e., ${S}_{i(i+\mathrm{1)...(}i-\mathrm{1)}}^{j}$), for $j$ = 1, 2, 3, …, $l$ + $k$ $l$, where ${\tilde{C}}_{i}^{j}$ = ${C}_{(i+\mathrm{1)}i}^{j}$ $+$ ${C}_{(i+\mathrm{2)}i}^{j}$ $+$…$+$ ${C}_{(i-\mathrm{1)}i}^{j}$. After that, he/she measures each of the $l$ + $kl$ processed photons in the original basis as he/she prepares it. That is, if the original state of the processed photon is in basis {$\mathrm{|0}\rangle$, $\mathrm{|1}\rangle$}/{$|+\rangle$, $|-\rangle$}, he measures it in basis {$\mathrm{|0}\rangle$, $\mathrm{|1}\rangle$}/{$|+\rangle$, $|-\rangle$}. In ideal condition, ${P}_{i}$ can obtain an ($l$ + $kl$)-bit string ${\bar{K}}_{i}$ = ${K}_{i+1}$ $\oplus$ ${K}_{i+2}$ $\oplus$…$\oplus$ ${K}_{i-1}$ according to the $l$ + $kl$ measurement results. To be specific, if the measurement result of the $j$-th photon is the same as (opposite to) its original state, ${\bar{K}}_{i}^{j}$ = 0 (1). Once getting ${\bar{K}}_{i}$, ${P}_{i}$ is able to deduce a ($l$ + $kl$)-bit string ${\hat{K}}_{i}$ = ${K}_{i}$ $\oplus$ ${\bar{K}}_{i}$ = ${K}_{1}$ $\oplus$ ${K}_{2}$ $\oplus$…$\oplus$ ${K}_{n}$. Just in the same manner, each participant gets his/her corresponding ($l$ + $kl$)-bit string, i.e., ${P}_{i}$ gets ${\hat{K}}_{i}$ for $i$ = 1, 2, 3, …, $n$. Obviously, in ideal condition, these $n$ binary strings should be identical if there exists no eavesdropping.

(6) The $n$ participants check eavesdropping with the $kl$ positions chosen in step (4) and the ($l$ + $kl$)-bit strings obtained in step (5). Specifically, each of the $n$ participants publishes the values of the $kl$ positions of his/her obtained string, i.e., ${P}_{i}$ publishes the corresponding $kl$ bits of ${\hat{K}}_{i}$ for $i$ = 1, 2, 3, …, $n$. Then each participants compares his/her announced $kl$-bit string with the ones published by the other participants. With the comparison results of the $kl$ positions, they can judge whether the procedure is secure. If there exists no eavesdropping, each participant ${P}_{i}$ drops the bits used for checking eavesdropping and getting an $l$-bit binary string with the remaining bits, i.e., ${\hat{\mathrm{K\text{'}}}}_{i}$, $i$ = 1, 2, 3, …, $n$; otherwise, they abort the protocol. In ideal condition, by utilizing steps (1)-(5), the $n$ established random strings should be identical, i.e., ${\hat{K}\text{'}}_{1}$ = ${\hat{K}\text{'}}_{2}$ = … = ${\hat{K}\text{'}}_{n}$. To check consistency of the $n$ binary strings, each participant ${P}_{i}$ calculates the hash value $h$($\lambda$ $\parallel$ ${\hat{K}\text{'}}_{i}$). Here, $h$: ${\mathrm{\{0,1\}}}^{\ast }$ $\to$ ${\mathrm{\{0,1\}}}^{s}$ is a one-way hash function previously chosen by the $n$ participants, and $\lambda$ = $f({\lambda }_{1},{\lambda }_{2},\,\mathrm{...,}{\lambda }_{n})$ (e.g., $\lambda$ = $f({\lambda }_{1}$ $\oplus$ ${\lambda }_{2}$ $\oplus$… $\oplus$ ${\lambda }_{n}$), where ${\lambda }_{i}$ is a random bit generated and announced by ${P}_{i}$ after deducing ${\hat{K}\text{'}}_{i}$, $i$ = 1, 2, …, $n$. If all the $n$ hash values are identical, the $n$ participants have established a common key $K$ = ${\hat{K}\text{'}}_{1}$ = ${\hat{K}\text{'}}_{2}$ = … = ${\hat{K}\text{'}}_{n}$; otherwise, they abort the results and restart the protocol.

Thus far, we have presented a new efficient travelling-mode MQKA protocol. In practical situation, there may exist a certain number of errors, which are caused by the noise, in ${\hat{K}\text{'}}_{i}$, $i$ = 1, 2, …, $n$. And this may lead the protocol fails in step (6) with a high probability. To circumvent this problem, several existing methods, such as quantum error correction codes (QECC)^69^ and quantum error avoiding codes (QEAC)^70^ could be utilized. Moreover, as the proposed MQKA protocol contains two-way quantum communication, the participants involved in the protocol should also make use of a filter and a beam splitter to prevent the Trojan horse attack and the invisible-photon attack in practical implementation^71,72^.

### Security and fairness analysis

Herein we analyze the security and fairness of the proposed MQKA protocol. We first demonstrate that the protocol can achieve security property, i.e., be secure against the attacks from the external eavesdropper. Then we show its fairness property, i.e., be immune to the attacks from dishonest participants.

### Security analysis

To analyze the security of the proposed protocol, we first suppose that Eve is an evil attacker who wants to eavesdrop the final common key without being noticed by the legal participants. Based on the principles of the proposed MQKA protocol, if Eve wants to achieve this goal, she should be capable of obtaining the private key of each participant without being found. To get a participant’s private key, Eve can make use of different kinds of attacks. For instance, she could intercept and substitute the travelling photons, which are sent to the legal receiver, with the ones prepared by herself, or she could entangle the travelling photons with some additional states, with which she may be able to extract some valuable information about the encoded private key. However, in the proposed protocol, the private keys and controlling strings of the participants are encoded on the transmitted photons by performing certain unitary operations. Hence, whatever kind of attack Eve utilizes, the action to eavesdrop a participant’s private key is equivalent to discriminate the operations that he/she has performed on the transmitted photon sequences. For example, when ${P}_{i}$ receives the photon sequence from ${P}_{i-1}$ in Round $v$, $v$ $\in$ {1, 2, 3, …, $n$−1}, he/she encodes ${K}_{i}^{j}$ and ${C}_{i(i+n-v)}^{j}$(=${C}_{i(i-v)}^{j}$) onto the $j$-th photon of the received sequence ${S}_{(i-v)(i-v+\mathrm{1)...(}i-\mathrm{1)}}$. Obviously, if Eve wants to get ${K}_{i}^{j}$, she should know which one of the operations, $I$, $F$, $W(\frac{p\pi }{n})$ and $F$ $W(\frac{p\pi }{n})$), ${P}_{i}$ has performed on the corresponding photon. Since the controlling bit ${C}_{i(i-v)}^{j}$ will be encoded only once, Eve should be capable of discriminating the four unitary operations with a single use. In fact, these four unitary operations, $I$, $F$, $W(\frac{p\pi }{n})$ and $F$ $W(\frac{p\pi }{n})$, utilized our protocol cannot be unambiguously discriminated with a single use. To demonstrate this conclusion, we first introduce a theory on quantum operation discrimination.

#### **Theorem 1**

 The quantum operations ${\zeta }_{1}$, …, ${\zeta }_{n}$ can be unambiguously discriminated by a single use if and only if for any $i$ = 1, 2, …, $n$, supp(${\zeta }_{i}$) $\nsubseteq$ supp(${M}_{i}$), where supp($\zeta$) denotes the support of a quantum operation $\zeta$ and ${M}_{i}=\{{\zeta }_{j}:j\ne i\}$ ^73^.

It is easy to find that the unitary operations, $I$, $F$, $W(\frac{p\pi }{n})$ and $F$ $W(\frac{p\pi }{n})$, satisfy the following relationship,

4 $$W(\frac{p\pi }{n})=\,\cos (\frac{p\pi }{2n})\cdot I+\,\sin (\frac{p\pi }{2n})\cdot F+0\cdot FW(\frac{p\pi }{n}),$$

which indicates that supp($W(\frac{p\pi }{n})$) $\subseteq$ supp($I$, $F$, $FW(\frac{p\pi }{n})$). Hence according to according to Theorem 1, these four operations cannot be unambiguously discriminated by a single use. Namely, when these four unitary operations are respectively performed on a single qubit or one qubit of any entangled state, they cannot be unambiguously discriminated.

Moreover, in practical implementation, the participants involved in this protocol will make use of the methods given in refs^71,72^ to resist the Trojan horse attack and invisible photon attack in each transmission of the every photon sequence. Therefore, the protocol is also immune to these two attacks.

### Fairness analysis

It is well known that, for a multiparty quantum cryptographic protocol, the participant attacks^74^ (i.e., the attacks from dishonest participants) are always more threatening than external attacks (i.e., the attacks from external eavesdroppers). In the executing process of a multiparty protocol, a dishonest participant has more opportunities to attack the protocol. First, he/she is able to replace the legal photon sequences, which are prepared or received by himself/herself, with whatever he/she wants. Second, in order to avoid introducing errors into the eavesdropping check, he/she could tell lies in the phase of classical information exchange. More important, in order to occupy a greater advantage, some dishonest participants can collaborate to cheat in the executing process of the protocol. Now, we make an analysis on the fairness of the proposed protocol to show its immunity to the participant attacks.

In the participant attack on the proposed protocol, one or more dishonest participants try to predetermine the final common key without being found by the honest participant. To show the immunity of the proposed protocol to such kind of attack, we consider the worst case, where the number of the honest participants is 1. In other words, $n$-1 dishonest participants collaborate to determine the final common key. Obviously, if the proposed protocol is immune to the participant attack under this assumption, it can also resist the participant attacks where the number of the dishonest participants is less than $n$−1.

Herein we show the immunity of the proposed protocol to the worst case. Without loss of generality, we suppose that ${P}_{i}$ is the only participant who is honest, $i$ $\in${1, 2, …, $n$}. According to the principles of the proposed protocol, we can find out that the final key gotten by ${P}_{i}$ (i.e., ${\hat{K}\text{'}}_{i}$) is part of the ($l$ + $kl$)-bit string ${\hat{K}}_{i}$, $i$ $\in${1, 2, …, $n$}. Hence, if the $n$−1 dishonest participants could predetermine ${\hat{K}}_{i}$, they will have significant advantage in determining ${\hat{K}\text{'}}_{i}$. Apparently, the key point for the $n$−1 dishonest participants to predetermine ${\hat{K}}_{i}$ is to eavesdrop ${P}_{i}$’s private key ${K}_{i}$ before ${S}_{i(i+\mathrm{1)...(}i-\mathrm{1)}}$ is sent back to ${P}_{i}$. For example, if the $n$−1 dishonest participants have obtained ${K}_{i}$ and want ${\hat{K}}_{i}$ to be ${K}^{\ast }$, they can attack as follows. When ${P}_{i+j}$ receives the sequence generated by ${P}_{i}$, i.e., ${S}_{i(i+\mathrm{1)..(}i+j-\mathrm{1)}}$, for $j$ = 1, 2, …, $n$-2, he/she only encodes his/her corresponding controlling string ${C}_{(i+j)i}$ onto ${S}_{i(i+\mathrm{1)..(}i+j-\mathrm{1)}}$. This is equivalent to the case that ${K}_{i+j}$ is a zero vector. After receiving ${S}_{i(i+\mathrm{1)..(}i-\mathrm{2)}}$ from ${P}_{i-2}$, ${P}_{i-1}$ encodes ${K}_{i}$ $\oplus$ ${K}^{\ast }$ and ${C}_{(i-\mathrm{1)}i}$ onto ${S}_{i(i+\mathrm{1)..(}i-\mathrm{2)}}$. Then he/she sends the sequence ${S}_{i(i+\mathrm{1)..(}i-\mathrm{1)}}$ to ${P}_{i}$. Obviously, if there exists no external eavesdropping and the $n$−1 dishonest participants honestly announces their controlling strings in step (5). The ($l$ + $kl$)-bit string obtained by ${P}_{i}$ is ${K}^{\ast }$, i.e., ${\hat{K}}_{i}$ = ${K}^{\ast }$ $\oplus$ ${K}_{i}$ $\oplus$ ${K}_{i}$ = ${K}^{\ast }$.

As we analyzed in security analysis, the bits of ${K}_{i}$ and the corresponding controlling string are encoded onto the received photon sequence with the four unitary operations, $I$, $F$, $W(\frac{p\pi }{n})$ and $F$ $W(\frac{p\pi }{n})$. In other words, no matter what kind of attacking strategy the dishonest participants utilize, if they want to get ${K}_{i}$ before being aware of ${P}_{i}$’s controlling strings, they should be capable of unambiguously discriminating the four unitary operations with a single use. Nevertheless, according to the conclusion given above, the dishonest participants can never have this ability since these four unitary operations cannot be unambiguously discriminated with a single use. For instance, according to the principles of the proposed protocol, if the $j$-th photon of a sequence sent from ${P}_{i-1}$, i.e., ${S}_{i-1}^{j}$, is in basis {$\mathrm{|0}\rangle$, $\mathrm{|1}\rangle$}($|+\rangle$, $|-\rangle$}), after ${P}_{i}$’s processing, the $j$-th photon of the sequence ${S}_{(i-\mathrm{1)}i}$, i.e., ${S}_{(i-\mathrm{1)}i}^{j}$, will randomly be one of the state in {$\mathrm{|0}\rangle$, $\mathrm{|1}\rangle$, $|\bar{0}\rangle$, $|\bar{1}\rangle$} ({$|+\rangle$, $|-\rangle$, $|\bar{+}\rangle$, $|\bar{-}\rangle$}, where

5 $$\begin{array}{c}|\bar{0}\rangle =\,\cos (\frac{p\pi }{2n})\mathrm{|0}\rangle +\,\sin (\frac{p\pi }{2n})\mathrm{|1}\rangle ,\\ |\bar{1}\rangle =\,\cos (\frac{p\pi }{2n})\mathrm{|1}\rangle -\,\sin (\frac{p\pi }{2n})\mathrm{|0}\rangle ,\\ |\bar{+}\rangle =\,\cos (\frac{p\pi }{2n})|+\rangle -\,\sin (\frac{p\pi }{2n})|-\rangle ,\\ |\bar{-}\rangle =\,\cos (\frac{p\pi }{2n})|-\rangle +\,\sin (\frac{p\pi }{2n})|+\rangle .\end{array}$$

Obviously, without the controlling bit ${C}_{i(i-\mathrm{1)}}^{j}$, ${P}_{i+1}$ is unable to correctly deduce ${K}_{i}^{j}$ since he/she does not know which basis he can utilize to measure ${S}_{(i-\mathrm{1)}i}^{j}$. In fact, according the the conclusion drawn above, no matter what kind of state ${S}_{(i-\mathrm{1)}}^{j}$ is in, ${P}_{i+1}$ cannot deduce ${K}_{i}^{j}$ correctly.

Based on the above analysis, we have shown that the dishonest participants are unable to get ${K}_{i}$ before ${P}_{i}$ receives ${S}_{i(i+\mathrm{1)...(}i-\mathrm{1)}}$. More precisely, the dishonest participants are unable to get ${K}_{i}$ before ${P}_{i}$ publishes his/her controlling strings. Under this circumstance, to eavesdrop ${K}_{i}$, each of the dishonest participants has to process the photon sequence, which is generated by ${P}_{i}$, strictly following the principles of the proposed protocol. It is not hard to find that, by utilizing this strategy, the dishonest participants could deduce ${K}_{i}$ with the photon sequences prepared by themselves and the controlling strings announced by ${P}_{i}$. After obtaining ${K}_{i}$, the dishonest participants can deduce ${\hat{K}}_{i}^{\text{'}}$, which will be obtained by ${P}_{i}$ in step (6), before announcing their controlling own strings. However, once the protocol proceed to this step, the only method that they can use to modify ${\hat{K}}_{i}^{\text{'}}$, is to announce fake controlling strings. Concretely, if the dishonest participants want to modify the $j$-th bit of ${\hat{K}}_{i}^{\text{'}}$, i.e., ${\hat{K}}_{i}^{\text{'}j}$, they should change the values of their corresponding controlling bits, ${C}_{(i+\mathrm{1)}i}^{j}$, …, ${C}_{(i-\mathrm{1)}i}^{j}$, to make ${P}_{i}$ perform an additional operation $F$ on ${S}_{i(i+\mathrm{1)...(}i-\mathrm{1)}}^{j}$. However, whatever strategy they employ to modify the controlling bits that will be announced, the additional unitary operation, which they can make ${P}_{i}$ perform on ${S}_{i(i+\mathrm{1)...(}i-\mathrm{1)}}^{j}$, will ineluctably belong to $\bar{W}$ = $\{W(\frac{-p\pi }{n})$, $W(\frac{-2p\pi }{n})$, …, $W(\frac{-(n-\mathrm{1)}p\pi }{n})\}$. It is easy to verify that, no matter what operation in $\bar{T}$ is performed on ${S}_{i(i+\mathrm{1)...(}i-\mathrm{1)}}^{j}$, the final state of ${S}_{i(i+\mathrm{1)...(}i-\mathrm{1)}}^{j}$ (i.e., the state of ${S}_{i(i+\mathrm{1)...(}i-\mathrm{1)}}^{j}$ after being performed $W(\frac{-{\tilde{C}}_{i}^{j}p\pi }{n})$ on it) will be transformed to a superposition state of $\mathrm{|0}\rangle$ and $\mathrm{|1}\rangle$ ($|+\rangle$ and $|-\rangle$), which indicates that the $n$−1 dishonest participants are unable to deterministically determine the value of ${\hat{K}}_{i}^{\text{'}j}$. Till now, we have shown that the proposed protocol is immune to the participant attack.

## Discussion

Thus far, an efficient travelling-mode MQKA protocol has been proposed. In order to illustrate the advantages of this protocol, a discussion, where the proposed protocol is compared with the existing MQKA protocols, is made first in this section. After that, we end this paper with a short conclusion.

### Key indicators of the proposed protocol

Before comparing the proposed protocol with the existing MQKA protocols, we first focus on some key indicators of the proposed protocol, i.e., qubit efficiency, measurement efficiency and the unitary operation efficiency.

Firstly, to check eavesdropping, the proposed protocol only need the $n$ participants cooperate to perform one eavesdropping detection, i.e, the detection in step (6). In other words, in the process of the photon sequence transmitting, i.e., in steps (1)–(5), a participant need not perform any eavesdropping detection when they received a photon sequence from his/her previous participant. Obviously, this is the main merit of the proposed protocol since it can greatly reduce the number of the photons used for checking eavesdropping, and hence make the proposed efficient, i.e., has a high qubit efficiency. The qubit efficiency here is defined as $\eta$ = ${n}_{c}$/${n}_{q}$, where ${n}_{c}$ is the length of the final common key established in this protocol, and ${n}_{q}$ is the total number of the photons used for establishing the corresponding final common key. Concretely, to establish an $l$-bit final common key in ideal condition, each of the involved participants should prepare a sequence of $l$ + $kl$ photons. In the only one eavesdropping detection, each participant will use $kl$ photons in his/her sequences for checking eavesdropping. Since there are $n$ participants involved in the proposed protocol, the total number of the photons, which will be used in establishing an $l$-bit final common key, is $n(l+kl)$. Therefore, the qubit efficiency of the proposed protocol is

6 $$\frac{l}{n(l+kl)}=\frac{1}{n\mathrm{(1}+k)}.$$

Secondly, as the proposed protocol only need one eavesdropping detection, the number of the measurements required in this protocol is relatively small. Specifically, to establish an $l$-bit final common key, each of the participants need perform $l$ + $kl$ measurements in theory. Namely, ($l$ + $kl$)$n$ measurements are needed in this whole procedure of the protocol. Hence, the measurement efficiency (the ratio of the length of final common key to the number of the performed measurements) of the protocol is

7 $$\frac{l}{(l+kl)n}=\frac{1}{n\mathrm{(1}+k)}.$$

Thirdly, since the security of the protocol is mainly based on the unitary operations performed on the transmitted photons. Here we calculate the unitary operation efficiency (the ratio of the length of final common key to the number of the performed unitary operations) of the protocol. Concretely, to establish an $l$-bit final common key, each of the participants need perform $n$($l$ + $kl$) unitary operations in theory. That is to say, ($l$ + $kl$)${n}^{2}$ unitary operations are needed in total. Thus, the unitary operation efficiency of the proposed protocol is

8 $$\frac{l}{(l+kl){n}^{2}}=\frac{1}{\mathrm{(1}+k){n}^{2}}.$$

Moreover, in the existing MQKA protocols, after the participants confirm that there exists no eavesdropping in the executing procedure of the protocol, each participant directly makes use of the measurements results of the remaining quantum information carriers to deduce a binary string as his/her final key. However, they do not check whether the keys in their hands are identical. In order to solve this problem, we have added a step, i.e., step (6), in this proposed to check the consistency of the final keys in the participants’ hands.

### Comparison

Herein we compare the proposed protocols with seven existing MQKA protocols in the following five aspects: qubit efficiency, measurement efficiency, unitary operation efficiency, security against participant attack and key consistency check. The seven existing protocols are SZ13 protocol^55^, LGHW13 protocol^56^, SZWLL13 protocol^57^, SYW16 protocol^60^, SZWYZL16 protocol^61^, SHW15 protocol^64^, HSXLFJY16 protocol^67^ and HM17 protocol^68^. The indicators of these existing MQKA protocols are calculated below, and the specific comparison results are shown in Table 1 and Table 2.

**Table 1:** Comparison of the efficiencies. In this table, ${\eta }_{q}$, ${\eta }_{m}$ and ${\eta }_{u}$ are qubit efficiency, measurement efficiency and unitary operation efficiency, respectively. The number in [] represents the concrete value of the efficiency when $n$ = 10 and $k$ = 0.5.

*n*-party protocols	${{\boldsymbol{\eta }}}_{{\boldsymbol{q}}}$	${{\boldsymbol{\eta }}}_{{\boldsymbol{m}}}$	${{\boldsymbol{\eta }}}_{{\boldsymbol{u}}}$
SZ13 protocol^55^	$\frac{1}{\mathrm{(2}+kn)n}$; $\frac{1}{\mathrm{[70]}}$	$\frac{2}{\mathrm{(2}+k){n}^{2}}$; $\frac{1}{\mathrm{[125]}}$	0
LGHW13 protocol^56^	$\frac{1}{(n-\mathrm{1)(1}+k)n}$; $[\frac{1}{135}]$	$\frac{1}{(n-\mathrm{1)(1}+k)n}$; $[\frac{1}{135}]$	0
SZWLL13^57^	$\frac{1}{\mathrm{(1}+kn)n}$; $[\frac{1}{60}]$	$\frac{1}{\mathrm{(1}+kn)n}$; $[\frac{1}{60}]$	$\frac{1}{{n}^{2}}$; $[\frac{1}{100}]$
SYW16 protocol^60^	$\frac{2}{\mathrm{(4}+kn)n}$; $[\frac{1}{45}]$	$\frac{2}{(kn+\mathrm{1)}n}$; $[\frac{1}{30}]$	$\frac{2}{(n-\mathrm{1)}n}$; $[\frac{1}{45}]$
SZWYZL16 protocol^61^	$\frac{1}{(3+2kn)n}$; $[\frac{1}{130}]$	$\frac{2}{(3+4kn)n}$; $[\frac{1}{115}]$	$\frac{2}{(2{\rm{n}}-1)n}$; $[\frac{1}{95}]$
SHW15 protocol^64^	$\frac{1}{\mathrm{(1}+kn)n}$; $[\frac{1}{60}]$	$\frac{1}{\mathrm{(1}+kn)n}$; $[\frac{1}{60}]$	$\frac{1}{\mathrm{(1}+n)n}$; $[\frac{1}{110}]$
HSXLFJY16 protocol^67^	$\frac{1}{\mathrm{(1}+kn)n}$; $[\frac{1}{60}]$	$\frac{1}{\mathrm{(1}+kn)n}$; $[\frac{1}{60}]$	$\frac{1}{{n}^{2}}$; $[\frac{1}{100}]$
CM17 protocol^68^	$\frac{1}{\mathrm{(1}+kn)n}$; $[\frac{1}{60}]$	$\frac{1}{\mathrm{(1}+kn)n}$; $[\frac{1}{60}]$	$\frac{2}{(n+\mathrm{1)}n}$; $[\frac{1}{55}]$
Our protocol	$\frac{1}{n\mathrm{(1}+k)}$; $[\frac{1}{15}]$	$\frac{1}{n\mathrm{(1}+k)}$; $[\frac{1}{15}]$	$\frac{1}{\mathrm{(1}+k){n}^{2}}$; $[\frac{1}{150}]$

**Table 2:** Comparison of the other indicators.

${\boldsymbol{n}}$-party protocols	key consistency check	security against participant attack	needing entanglement?
SZ13 protocol^55^	No	insecure	Yes
LGHW13 protocol^56^	No	secure	No
SZWLL13^57^	No	insecure	No
SYW16 protocol^60^	No	insecure	Yes
SZWYZL16 protocol^61^	No	insecure	Yes
SHW15 protocol^64^	No	insecure	No
HSXLFJY16 protocol^67^	No	secure	No
CM17 protocol^68^	No	secure	Yes
Our protocol	Yes	secure	No

#### SZ13 protocol

This protocol make use of entanglement to establish the final common key, and utilize the technique of decoy particles to assure its security. The qubit efficiency of this protocol is $\frac{1}{\mathrm{(2}+kn)n}$. The measurement efficiency of this protocol is $\frac{2}{\mathrm{(2}+k){n}^{2}}$. It should be pointed out that the measurements performed in this protocol includes both single-particle measurements and multi-particle measurements (i.e., Bell measurements). More important, this protocols is vulnerable to the participant attack^55,56^.

#### LGHW13 protocol

The protocol is immune to the participant attack, and it does not need utilize entanglement to establish the final key^56^. However, this protocol is quite inefficient due to a low qubit efficiency $(\frac{1}{(n-\mathrm{1)(1}+k)n})$ and a low measurement efficiency $(\frac{1}{(n-\mathrm{1)(1}+k)n})$.

#### SZWLL13

This protocol attempts to establish a final common key efficiently with single photons and unitary operations. The qubit efficiency, measurement efficiency and unitary efficiency of this protocol are $\frac{1}{\mathrm{(1}+kn)n}$, $\frac{1}{\mathrm{(1}+kn)n}$ and $\frac{1}{{n}^{2}}$, respectively. However, it cannot stand against the participant attack^57,58^.

#### SYW16 protocol

The protocol tries to achieve the purpose of MQKA with four-qubit entangled cluster states. Its qubit efficiency, measurement efficiency and unitary efficiency are $\frac{2}{\mathrm{(4}+kn)n}$, $\frac{2}{(kn+\mathrm{1)}n}$ and $\frac{2}{(n-\mathrm{1)}n}$, respectively. It should be noticed that the cluster basis measurements performed in this protocol is more difficult to implement than single-particle measurements under the current techniques. Moreover, this protocol is susceptible to the participant attack^60,65^.

#### SZWYZL16 protocol

Due to the utilizing six-qubit entangled states, this protocol is not very efficient. Concretely, the qubit efficiency, measurement efficiency and unitary operation efficiency are $\frac{1}{\mathrm{(3}+2kn)n}$, $\frac{2}{\mathrm{(3}+4kn)n}$ and $\frac{2}{\mathrm{(2}n-\mathrm{1)}n}$, respectively. Moreover, this protocol is also insecure against participant attack^61,65^.

#### SHW15 protocol

This protocol aims to achieve an efficient MQKA with single photons and unitary operations. In fact, the qubit efficiency, measurement efficiency and unitary operation efficiency of this protocol are $\frac{1}{\mathrm{(1}+kn)n}$, $\frac{1}{\mathrm{(1}+kn)n}$ and $\frac{1}{\mathrm{(1}+n)n}$, respectively. However, this protocol still cannot be immune to the participant attack^64,65^.

#### HSXLFJY16 protocol

This protocol is presented with single photons, single-particle measurements and unitary operations^67^. Since the eavesdropping detection is needed is each step of the sequence transmission, The qubit efficiency, measurement efficiency and unitary operation efficiency are respectively $\frac{1}{\mathrm{(1}+kn)n}$, $\frac{1}{\mathrm{(1}+kn)n}$ and $\frac{1}{{n}^{2}}$.

#### CM17 protocol

This protocol is proposed with quantum search algorithm, where entanglement and unitary operations are needed^68^. The qubit efficiency, measurement efficiency and unitary operation efficiency are $\frac{1}{\mathrm{(1}+kn)n}$, $\frac{1}{\mathrm{(1}+kn)n}$ and $\frac{2}{(n+\mathrm{1)}n}$.

## Conclusion

In this paper, we focus on improving the efficiency of the travelling-mode MQKA protocol and propose an efficient MQKA protocol with single photons. Security and fairness analysis shows that the proposed protocol is immune to both the external attack and participant attack. By utilizing the ideas of collective eavesdropping, the qubit efficiency and measurement efficiency of this protocol are higher than those of the existing protocols, especially the ones which are also secure and fair. In addition, due to the utilization of single photons and single-particle measurement, the proposed protocol is more feasible with the current technologies than the ones which employ entanglement or multi-particle measurements.

Finally, we should point out that, to design a really practical QKA protocol, one should not only consider the fairness in the quantum exchange process (i.e., the process to generate raw keys), but also propose qualified information reconciliation process and privacy amplification process which can be utilized in QKA protocols for negotiating the final key fairly. How to design a qualified classical postprocessing processes for QKA/MQKA, still remains an open problem, which we would like to research in future.
